# Antimicrobial Activity of Lactic Acid Bacteria in Dairy Products and Gut: Effect on Pathogens

**DOI:** 10.1155/2015/584183

**Published:** 2015-03-16

**Authors:** Juan L. Arqués, Eva Rodríguez, Susana Langa, José María Landete, Margarita Medina

**Affiliations:** Departamento Tecnología de Alimentos, Instituto Nacional de Investigación y Tecnología Agraria y Alimentaria (INIA), Carretera de La Coruña km 7, 28040 Madrid, Spain

## Abstract

The food industry seeks alternatives to satisfy consumer demands of safe foods with a long shelf-life able to maintain the nutritional and organoleptic quality. The application of antimicrobial compounds-producing protective cultures may provide an additional parameter of processing in order to improve the safety and ensure food quality, keeping or enhancing its sensorial characteristics. In addition, strong evidences suggest that certain probiotic strains can confer resistance against infection with enteric pathogens. Several mechanisms have been proposed to support this phenomenon, including antimicrobial compounds secreted by the probiotics, competitive exclusion, or stimulation of the immune system. Recent research has increasingly demonstrated the role of antimicrobial compounds as protective mechanism against intestinal pathogens and therefore certain strains could have an effect on both the food and the gut. In this aspect, the effects of the combination of different strains keep unknown. The development of multistrain probiotic dairy products with good technological properties and with improved characteristics to those shown by the individual strains, able to act not only as protective cultures in foods, but also as probiotics able to exert a protective action against infections, has gained increased interest.

## 1. Bacteriocins to Improve Dairy Products Safety

### 1.1. Bacterial Contamination in Dairy Products

Farmed animals represent a major reservoir of pathogens that can be transferred to milk. The predominant human bacterial pathogens that can potentially be transferred to milk include mainly* Listeria monocytogenes*,* Salmonella* spp.,* Staphylococcus aureus*, and pathogenic* Escherichia coli*. Raw milk provides a potential growth medium for the development of these bacteria [1]. Although pasteurization destroys potential pathogenic microorganisms, postpasteurization processing can lead to the recontamination of dairy products.

*L. monocytogenes* can cause illnesses extending from those with mild flu-like symptoms or gastroenteritis to more serious, potentially fatal conditions such as bacteraemia and meningitis and in pregnancy can cause preterm delivery, foetal loss, neonatal infection, or infant death. Between 1998 and 2008 in USA, at least 25% of reported outbreaks of listeriosis were of dairy origin [2].* Listeria* can contaminate the dairy environment from manure or improperly fermented silage and can be introduced in the human food supply chain. The control of this pathogen in the food industry remains a challenge because of its ubiquitous character and its ability to grow at low temperatures and to survive and persist even in hostile environments. Soft cheeses can support the growth of* Listeria* introduced after processing independently of the use of raw or pasteurized milk. Recalls of dairy products, mainly soft cheeses, contaminated with the pathogen are relatively frequent. Because of its high case-fatality rate, listeriosis is, after salmonellosis, the second most frequent cause of food-borne infection-related deaths in Europe [3].

*S. aureus* is a causative agent of bovine mastitis capable of producing thermostable enterotoxins. Food-borne illness due to* S. aureus* can cause abdominal cramps, nausea, vomiting, and diarrhoea [4].* S. aureus* is a common environmental microorganism which is found in raw milk [5]. Dairy products contain low levels of enterotoxigenic staphylococci. However, temperature abuse above 10°C and poor starter culture activity during fermentation are factors involved in dairy related outbreaks of staphylococcal intoxication [6].

*E. coli* O157:H7 is a Shiga toxin-producing* Escherichia coli* (STEC) serotype of high virulence (it can cause disease at a dose of 5–50 cells). The number of cases of severe disease caused by STEC in dairy products has remained quite low, probably thanks to the compliance with good hygienic practices at the farm level [1]. The main reservoirs of STEC are ruminants, contaminating milk through subclinical mastitis or faecal routes, and the bacteria can persist in milking equipment. While severe cases of bloody diarrhoea or haemolytic uremic syndrome caused by STEC are uncommon, they do affect mostly vulnerable groups such as young children and elderly people.

*Salmonella* has long been recognized as an important human health problem of economic significance in animal and humans.* Salmonella* is found in the environment and in the gastrointestinal tract of farmed and wild animals. A total of 108,614 confirmed cases of salmonellosis were reported in the European Union in 2009, although cases attributed to* S. *enteritidis have decreased during the last years [7]. However,* Salmonella* infections have not declined over the past 15 years in USA [8]. Dairy products along with meat and eggs are the most common causes of food-borne infection by* Salmonella*. Salmonellosis from contaminated milk and dairy products has been associated with inadequate pasteurization and postprocess contamination. Most cheeses, including raw or pasteurized milk cheeses, properly manufactured and aged, appear to pose no significant health risk of* Salmonella* infection.

Several factors can increase the risk of food-borne infections and the severity of the diseases, resulting in a reduction in the number of pathogens needed to cause the disease and increased severity. The occurrence of infection in groups of people with a high risk was reviewed by Lund and O'Brien [9]. Susceptible population accounts for 15–20% of the general population in developed countries and includes mainly people with immunodeficiency, pregnant women, children, and the elderly. The risk of food-borne disease should be minimised for these vulnerable groups.

Many control measures in the food industry are provided to prevent or minimise bacterial contamination, including the appearance or growth of food-borne pathogens. Good manufacturing practices, sanitation, and hygiene measurements for raw material, the food industry environment, and so forth do not avoid the occurrence of food-borne outbreaks.

### 1.2. Applications of Bacteriocins and Bacteriocinogenic Strains in Dairy Products

The application of antimicrobial-producing lactic acid bacteria (LAB) or food-grade ferments in the manufacture of dairy products, which can be incorporated into fermented or nonfermented dairy products, implies a processing additional advantage to improve the safety and increase the quality of dairy products, providing an additional hurdle to reduce the likelihood of food-borne diseases (Table 1).

Bacteriocins are ribosomally synthesized bioactive peptides produced by bacteria displaying antimicrobial activity against related (narrow spectrum) or nonrelated (broad spectrum) bacteria. These peptides are considered natural biopreservatives and their potential application in the food industry has received great interest. On the basis of modifications of their precursor peptides, bacteriocins are classified into class I and class II [21]. Class I bacteriocins or lantibiotics undergo posttranslational modifications which introduce the thioether amino acids: lanthionine and methyllanthionine. Novel bacteriocins with translational modifications atypical of lantibiotics have been recently identified [22]. Class II contain unmodified peptides and are subdivided into four groups [23]: IIa (one-peptide pediocin-like bacteriocin), IIb (two-peptide bacteriocins), IIc (cyclic bacteriocins), and IId (linear non-pediocin-like one-peptide bacteriocins).

Bacteriocins are active against Gram-positive pathogens such as* L. monocytogenes* and* S. aureus* and may be effective against Gram-negatives if the outer membrane is destabilized [24]. Bacteriocins produced* in situ* through the incorporation of producing strains as starters or adjunct cultures in fermented dairy products can be applied to improve the safety of the product. The generally recognized as safe (GRAS) bacteriocin nisin produced by* Lactococcus lactis* was the first antibacterial peptide described in LAB. Nisin and pediocin PA1 are used in biopreservation, and preparations of these bacteriocins are applied commercially. The use of ferments or bacteriocin-producing starter cultures does not require regulatory approval or label declarations and is frequently considered a more attractive strategy to incorporate bacteriocins in foods [25, 26]. The efficacy of bacteriocins used in combination with other antimicrobial treatments or hurdles increasing the opportunity to target Gram-negative pathogens has been summarized [27], where synergistic antimicrobial effects have been demonstrated.

The earliest application of nisin in dairy products was the prevention of spoilage by clostridial species responsible for the late-blowing defect in cheese [28]. Nisin was bactericidal against different strains of* L. monocytogenes*, and its effect was enhanced by addition of NaCl or reduction of pH [29]. Other lantibiotics have been applied in the elimination of* L. monocytogenes* in dairy products. The broad spectrum lacticin 3147 powder produced by* Lc. lactis* DPC 3147 inactivated this pathogen in yogurt and in cottage cheese [10].

Due to its strong antilisterial activity and its stability and activity in a wide range of pH values, pediocin has been applied in dairy products. A dried preparation of pediocin decreased* L. monocytogenes* counts in cottage cheese, cream, and cheese sauce systems [11], although the pathogen restarted growth in the mildly acidic and neutral food systems. Regrowth of* L. monocytogenes* also occurred with piscicolin 126 a class IIa produced by* Carnobacterium piscicola* JG126 in Camembert cheese [12]. Cell-free preparations of enterocins as enterocin CRL 35 reduced* Listeria* up to 9 log units in goat cheese at the end of the ripening period [13].

As direct addition of bacteriocins to food systems could result in some loss of the antimicrobial activity due to the diffusion into the food matrix or the interaction with food components, different strategies of incorporation have been considered. Microencapsulation of bacteriocins in liposomes has been proposed as an alternative to the direct addition of free bacteriocin to milk to improve stability and distribution in cheese, while preventing the antimicrobial action on the cheese starter during manufacture [30]. Nisin was encapsulated in nanovesicles from soy lecithin and inactivated* L. monocytogenes* growth in milk at low temperatures over 14 d, being as effective as free nisin [31]. Bioactive packaging with bacteriocins incorporated in different films was applied in sliced Cheddar cheese. Nisin in cellulose-based bioactive inserts reduced levels of* Listeria innocua* and* S. aureus* by approximately 2 logs during storage in modified atmosphere packaging (MAP) at refrigeration temperatures [32]. Sorbitol-plasticized sodium caseinate films containing nisin also reduced* L. innocua* counts on surface inoculated cheese by approximately 1 log unit. Although nisin did not migrate much inside the cheese matrix, films were effective against surface contaminated cheese [33].

Bacteriocinogenic cultures as starter or adjunct cultures in cheese manufacture permit the bacteriocin production* in situ*, reduce the cost of the biopreservation, and do not require regulatory approval. Nisin-producing strains in combination with other nisin resistant or tolerant cultures with desirable properties have been proposed as an alternative to the addition of nisin in commercial form. Selected mixed starter cultures with a nisin Z-producing* Lc. lactis* subsp.* lactis* biovar diacetylactis strain and a commercial starter were successfully developed by Bouksaim et al. [34]. Nisin-producing suitable strains for cheese making have been isolated from natural environments as raw milk and raw milk cheese [35, 36]. Nisin-producing starter cultures inactivated* L. monocytogenes* in Camembert cheese, although regrowth of the pathogen occurred when pH increased in this cheese variety [14]. A decrease in* L. monocytogenes* counts was registered by Rodríguez et al. [15] when nisin-producing* Lc. lactis* subsp.* lactis* ESI 515 and TAB 50 were used as single-starter cultures in the manufacture of raw milk cheese.

Other bacteriocinogenic cultures have been assayed in cheese manufacture. In Cheddar cheese manufactured with lacticin 3147-producing cultures, the bacteriocin was stable over 6-month ripening [37]. Lacticin 3147-producing transconjugant* Lc. lactis* DPC 4275 strain used as starter culture in the manufacture of cottage cheese reduced numbers of* L. monocytogenes* to <10 cells/g within 5 d at 4°C [16]. Lacticin-481 producing* Lc. lactis* subsp.* cremoris* TAB 24 used as single-starter in cheese lead to counts of the pathogen 2.5 units lower than in cheese made with a commercial starter [15]. Nisin A, nisin Z, and lacticin-481 producing lactococci selected by their technological potential as starter cultures [38] were useful to control* L. monocytogenes* in cottage cheese, with a higher antilisterial activity with the nisin A producing strains.

Cell suspensions of pediocin-producing* Lactobacillus plantarum* WHE 92 sprayed on the surface of Munster cheese inhibited* L. monocytogenes* growth [19]. The production of pediocin in heterologous hosts is considered an alternative to extend the application of this bacteriocin in milk and dairy products. Pediocin-producing* Lc. lactis* MM 217 starter culture containing a plasmid coding the pediocin PA1 operon reduced* L. monocytogenes* levels in Cheddar cheese by 3 log units after 92 d of ripening [18]. Food-grade pediocin-producing lactococcal strains developed by Reviriego et al. [39, 40] and used as adjuncts to the starter culture reduced* L. innocua* counts in a cheese model system and* L. monocytogenes*,* S. aureus,* and* E. coli* O157:H7 in cheese [20]. Plantaricin 423-producing* Lb. plantarum* LMG P-26358 isolated from artisanal cheese and used as adjunct to a nisin-producing starter [41] was highly effective against* L. innocua* and compatible with nisin producers, showing interest in cheese technology.

Many enterococcal bacteriocins are class II pediocin-like bacteriocins with strong antilisterial activity. Their utilization in foods would require a case-by-case evaluation of safety of each potential strain [42]. Enterocin AS-48 has an important potential as biopreservative [43]. Enterocin AS-48-producing* Enterococcus faecalis* used as starter or coculture with a commercial lactic starter in the manufacture of raw milk Manchego cheese decreased* L. monocytogenes* counts by 6 log units after 7 d [17] and completely inactivated the pathogen during the manufacture and ripening of raw milk cheese manufactured without starter culture [15].

Combinations of different preservation methods may act synergistically or provide higher protection than a single method alone. Bacteriocins have been combined with physical or biological treatments to allow the use of lower concentrations or reduce the severity of physical treatment, while achieving a higher lethality. Lacticin 3147 activity increased considerably after pressurization in skim milk or whey at 400–800 MPa [44], and the combination of this bacteriocin with 250 MPa acted synergistically lowering* S. aureus* counts in milk by more than 6 log units. Reductions of* S. aureus* in cheese by high pressure treatments combined with different bacteriocin-producing strains were synergistic [45]. This effect was also observed for* L. monocytogenes* [46] and* E. coli* O157:H7 [47]. Sublethal damage of the outer membrane of Gram-negatives or changes in membrane fluidity by pressurization could facilitate the access of bacteriocins to the cytoplasmic membrane.

Combinations of bacteriocins and reuterin, an antimicrobial compound produced by some strains of* Lb. reuteri*, exhibited a clear synergistic effect on the inhibition of* L. monocytogenes* and* S. aureus* in milk [48, 49]. Nisin did not inactivate five selected Gram-negative pathogens in milk [50], whereas reuterin reduced* E. coli* O157:H7,* S. *enteritidis,* Campylobacter jejuni*,* Aeromonas hydrophila*, and* Yersinia enterocolitica* counts. The combination of nisin and reuterin achieved reductions close to those obtained with only reuterin, without enhancing the antimicrobial effect of reuterin.

## 2. Bacteriocins in the Prevention and Reduction of Intestinal Pathogens

Gut microbiota play an essential role in digestion, metabolism, and immune function. Changes in the diversity and function of this ecosystem have been associated with a range of diseases including functional bowel disorders, inflammatory immune diseases, insulin resistance, and obesity and infectious diseases as the caused by* Clostridium difficile*. Dysbiosis as a result of antibiotics usage or the presence of different pathogenic organisms can be prevented or reduced by probiotics consumption.

Probiotics, or live microorganisms which when administered in adequate amounts, confer a health benefit on the host, can exert protective effect in the control of intestinal pathogens. Antimicrobial activity is considered a probiotic trait. Several proposals to explain this activity are the production of bacteriocins, competitive exclusion of the pathogen binding, competition for nutrients, or modulation of the immune system [51]. However, the mechanisms of action in the prevention of different gastrointestinal disorders are still poorly understood. Most probiotics applied in food products are lactic acid bacteria, mainly* Lactobacillus* and* Bifidobacterium*.

The role of bacteriocins within the gastrointestinal tract (GIT) on the prevalence of the producing strain and the microbial diversity and the survival of pathogens was reviewed by Dobson et al. [52]. Bacteriocins could contribute to probiotic functionality acting as colonizing peptides that facilitate the introduction or dominance of the bacteriocin-producing strain into the GIT niche. They may act as antimicrobial peptides directly killing other bacteria, as signalling peptides through quorum sensing and cross talk with bacterial communities or as signalling cells of the host immune system [52]. Bacteriocins can inhibit the invasion of competing or pathogen strains in the community or modulate the composition of the microbiota and the host immune system [53]. A review of recent* in vivo* studies on bacteriocin-based treatments of human and animal infections and the potential of bacteriocins in health was published by Hammami et al. [54].

### 2.1. Purified Bacteriocins in the GIT

Purified bacteriocins can be used in the treatment of pathogenic bacteria and may be employed as alternative to existing antibiotics, limited by the emergence of resistant pathogens and the damage of the human commensal microbiota. The spread of antibiotic resistance particularly in the hospital environments is a significant problem of healthcare and resistant pathogens to multiple antibiotics are a major challenge as antibiotics used to treat some pathogens are no longer effective. This consideration was reviewed by Cotter et al. [55].

Antimicrobial activity of nisin and lacticin 3147* in vivo* has been recently demonstrated in a murine infection model. Lacticin 3147 was subcutaneously administered to mice infected intraperitoneally (IP) with a luminescent* S. aureus* to analyze* in vivo* imaging. After 6 h of infection, photoluminescence and microbial analyses of liver, kidneys, and spleen revealed that the bacteriocin controlled the systemic spread of* S. aureus* in mice by preventing the dissemination of the pathogen [56]. Similar experiments were carried out by Campion et al. [57] with nisin A and its bioengineered variant with increased bioactivity nisin V [58] against bioluminescent* L. monocytogenes* EGDe in mice infected IP. Antimicrobial effect of nisin V was higher than the one observed with nisin A to control the infection with* L. monocytogenes* in mice, pointing to the interest in this peptide for therapeutic applications.

*C. difficile* can take profit from the antibiotic broad spectrum associated disruption of the microbiota and grow and produce toxins in the gut. Lacticin 3147 has the potential to be employed in the treatment of* C. difficile* diarrhoea and to eliminate the pathogen when added to an anaerobic fecal fermentation, although levels of the bacteriocin required were much higher than the antibiotic needed [59]. In the same way, other members of the GIT microbiota were affected by this application [59, 60].

The presence of nisin in duodenum, ileum, and faeces of rats treated with pure nisin was reported by Bernbom et al. [61], although nisin inactivation was registered when the concentrations estimated by ELISA were compared with a biological assay. These authors investigated the ability of pure nisin, a nisin-producing* Lc. lactis* CHCC 5826 and the isogenic non-nisin-producing* Lc. lactis* CHCC 2862 to modify the composition of the intestinal microbiota of human microbiota-associated rats. Both microbial cultures affected the composition of the intestinal microbiota increasing bifidobacteria levels and decreasing* Enterococcus/Streptococcus* populations in faeces, but the effect was not observed when purified nisin was administered.

Pediocin PA-1 producing strain* P. acidilactici* UL5 [62], able to inhibit* L. monocytogenes in vitro,* did not reduce the pathogen in the intestine of mice when administered intragastrically at high levels and was not detected in faeces. However, repeated doses of the purified pediocin PA-1 provided up to 2-log reductions in fecal listerial counts compared to the infected control group and slowed pathogen translocation into the liver and spleen, leading to the disappearance of* L. monocytogenes* infection in these two organs within six days. Pediocin PA-1 did not affect the composition of the mouse intestinal flora [62].

Bacteriocin-producing* Lb. salivarius* NRRL B-30514 or* Paenibacillus polymyxa* NRRL B-30509 inhibited* Campylobacter jejuni in vitro*, but the strains did not affect the pathogen in chickens. When the purified bacteriocin was encapsulated and administered to chickens colonized with the pathogen [63],* C. jejuni* was reduced by at least 6 log units. According to these authors, the bacteriocin was produced* in situ* in limiting quantities to kill* C. jejuni* when the strains were administered.

### 2.2. Bacteriocin-Producing Probiotics

The production of bacteriocins* in situ* by probiotics selected by their ability to survive in the GIT may be advantageous as proteolysis during gastric transit would be avoided. Although the protective effect of probiotics through bacteriocin production* in situ* has been studied, the determination of the fate of these peptides* in vivo* and the bacteriocin detection in complex environments present important limitations. Whereas studies detected the lack of efficacy* in vivo* of some bacteriocins, others provide evidence that bacteriocins can be produced and retain bioactivity in the GIT.

Although the lantibiotic lacticin 3147 was highly effective to inhibit pathogens, the producing lactococci were not able to confer protection against* L. monocytogenes* in a mouse model [64]. The bacteriocin-producing* Lc. lactis* DPC 6520 was able to survive the GIT passage in simulated conditions and* in vivo* survived the intestinal transit in mice and pigs, although the excretion rate was low (10^2^–10^5^ cfu/g) and the bacteriocin was not detected in faeces. When this strain was investigated against* C. difficile* in a simulated human distal colon using a bacteriocin negative variant as control, no reduction in the pathogen counts was registered. Previous data showed that lacticin 3147 delivered orally was rapidly degraded in the GIT [65].

Administration of human intestinal isolates pediocin PA1-producing* P. acidilactici* MM33 and nisin Z-producing* Lc. lactis* MM 19 increased total LAB and anaerobes in mice, and* P. acidilactici* also decreased Enterobacteriaceae levels. Both strains were resistant to acid and bile and reduced vancomycin resistant* Enterococcus* (VRE) intestinal colonization when administered orally with the two bacteriocin-producing cultures or the* P. acidilactici* M33A, a mutant without the capacity to produce bacteriocin. The eradication of VRE was attributed to pediocin activity as the pediocin negative derivative did not exhibit this antimicrobial effect against VRE [66].

Protective activity* in vivo* was not detected when pediocin AcH-producing* Lb. plantarum* DDEN 11007 or its nonproducing isogenic variant was studied [67] in gnotobiotic rats colonized with* L. monocytogenes*. Higher levels of the pathogen were detected in liver and spleen of animals colonized with the bacteriocin or the non-bacteriocin-producing strains. According to these authors, inoculating germ-free rats with the probiotic will induce immune responses facilitating* L. monocytogenes* to cross the epithelial barrier.

The antimicrobial activity of bacteriocin-producing probiotics in the GIT was observed with class II bacteriocin abp-118-producing* Lb. salivarius* UCC118 [68]. The administration of 10^9^ cfu/d during 3 days before infection reduced* L. monocytogenes* levels in mice compared with a variant bacteriocin-negative. The impact of this strain on the intestinal microbiota of mice and pigs was investigated by Riboulet-Bisson et al. [69].* Lb. salivarius* UCC118 or a mutant lacking bacteriocin production survived throughout the pig GIT and colonized the ileum. The bacteriocin-producing strain led to a significant decrease in Spirochaetes levels and affected Firmicutes genus members. This last effect was not observed when the mutant strain was administered and was thus associated with bacteriocin production.* Lb. salivarius* UCC118 administration has a significant but subtle impact on mouse and pig microbiota by a mechanism that seems, at least partially, bacteriocin-dependent.

At the GIT level, a probiotic mixture of* Lactobacillus* and* Pediococcus* of porcine intestinal origin alleviates* Salmonella* infection in a porcine model [70]. Salivaricin P-producing* Lb. salivarius* DPC6005, the only bacteriocin-producing strain in the mixture of probiotics administered to pigs, dominated over the rest of strains in the ileum digesta and mucosa. It was suggested that the predominance of this strain could be related to a competitive advantage attributed to bacteriocin production [71]. The increased efficacy of multistrain probiotics against pathogens may be caused by the greater variety of antimicrobial capacities associated with mixed preparations, such as production of weak organic acids, bacteriocins, hydrogen peroxide, coaggregation molecules and/or biosurfactants, and the stimulation of sIgA production and mucus secretion by the host [72]. According to Chapman et al. [73], multistrain probiotics show higher efficacy than single strains, although the studies published do not demonstrate whether synergistic interactions or higher probiotic doses are responsible for this effect.

Although production of bacteriocins by intestinal bacteria has been recognized, its prominent role within gut ecology has not been elucidated. In part, this could be due to the high metabolic costs expended by bacteria to elaborate and secrete these nonstructural polypeptides. It is likely that bacteriocins play additional roles in regulating the intestinal flora, such as signaling within and among microbial species.

Bacteriocins might act as quorum-sensing molecules or autoinducing peptides in the intestinal environment. Nisin acts as a secreted signal molecule that induces the transcription of the genes involved in its biosynthesis [74]. Cocultivation of* Lb. plantarum* DC400 with* Lb. sanfranciscensis* DPPMA174 leads to the induction of the synthesis of plantaricin A. As a response,* Lb. sanfranciscensis* increased the expression of proteins involved in stress response, amino acid metabolism, energy metabolism, membrane transport, nucleotide metabolism, and regulation of transcription [75]. Cultivation of* Lb. plantarum* DC400 with plantaricin A or with other lactobacilli increased the capacity to adhere to Caco-2 cells and to prevent the adhesion of potential intestinal pathogens. The adhesion or competition of* Lb. plantarum* DC400 was also mediated by the peptide plantaricin A and by cocultivation with other species in the ecosystem [76].

The specific probiotic cell products involved in immunomodulation are not well known. van Hemert et al. [77] studied a number of genes of* Lb. plantarum* that might influence the immune response of peripheral blood mononuclear cells, detecting specific genes encoding components of the plantaricin biosynthesis and transport pathway that might be responsible for the stimulation of anti- or proinflammatory immune responses in the gut. In fact, deletion of these genes from* Lb. plantarum* WCFS1 resulted in changes in IL-10 and IL-12 cytokine profiles compared with the wild type.

The identification of bacteriocin-producing potentially probiotic bacteria from the intestinal microbiota has been summarized by O'Shea et al. [22]. Considering the high proportion of intestinal bacteria that are nonculturable and the biased results of cultured-based screening procedures, emerging high throughput sequencing technologies and functional metagenomics-based approaches will be crucial to the identification of genes potentially encoding novel bacteriocins [22].

The effects of multistrain probiotics keep unclear. Although the number of studies is limited, multiple-strain cultures appear to exhibit greater efficacy than single strains, even when the strains are integrating the mixture. The development of multistrain probiotic dairy products with good technological properties, able to act as protective cultures in foods and as probiotics exerting a protective action against infections, has gained increased interest.

## 3. Future Trends

Bacteriocin effectiveness as biopreservatives in food may be hindered by the proteolytic activity of food or microbial enzymes, their adsorption to fat, and the appearance of resistant variants in sensitive strains. Food legislation for their approval and acceptance as food preservatives has also restricted their use, as only nisin and pediocin PA-1 are commercially available. In cheese manufacture, the activity of combined starters including both technological strains and bacteriocin-producing cultures is rather difficult to control for correct acidification, bacteriocin production, and quality of cheese. Compatible combinations of lactic starters and bacteriocin-producing strains may help to solve the problem. More research is needed for the optimization of bacteriocin production and activity in dairy products.

The simultaneous application of more than one bacteriocin or multiple bacteriocin producers may reduce the emergence of resistances in target strains. Bacterial cultures exhibiting overexpression of bacteriocins or multiple heterologous bacteriocin producers have received particular interest by researchers, although their industrial use would be limited by the restrictive legal regulations and the lack of acceptance by consumers. Combined treatments of bacteriocins with physical processes or other biopreservatives offer a wide scenario of practical future applications.

*In vitro* and animal studies have confirmed that the production of bacteriocins contributes to probiotic functionality in the GIT. The ability of a bacteriocin to function* in vivo* is influenced by the strain survival, the specific activity of the bacteriocin, the dosing regimen, the animal model, and the target organism. The factors controlling bacteriocin production in the GIT are not well understood and bacteriocin production in the GIT is difficult to assess. For that reason, standardized methods of assessing bacteriocin activity would be useful since variations in animal models, dosage, and quantification have made the comparison of data between laboratories difficult. This information will lead to human trials in which health properties will be accurately assessed.

The emergence of resistant pathogens is another area that deserves investigation. The application of bacteriocins in human health will depend on the knowledge of the mechanisms of action. The development of strategies for bacteriocin production at sufficient quantity and the performance of clinical trials to determine the efficacy of bacteriocins* in vivo* are areas that also would need to be addressed.

Multistrain probiotics appear to show higher efficacy than the single strains. Dairy products would be an effective vehicle for multistrain probiotic cultures, with good technological properties and improved characteristics to those shown by the individual strains, able to act not only as protective cultures in foods, but also as probiotic.

## Figures and Tables

**Table 1:** Applications of bacteriocins and bacteriocinogenic strains in dairy products.

Bacteriocin	Bacteriocin-producing culture	Application	Pathogen	Product	Reference
Lacticin 3147	*Lc. lactis *DPC 3147	Spray-dried powder	*L. monocytogenes *	Cottage cheese	[10]
Pediocin	*P. acidilactici *PAC1.0	Dry powder	*L. monocytogenes *	Cottage cheese and yogurt	[11]
Piscicolin 126	*C. piscicola* JG 126	Concentrated supernatant	*L. monocytogenes *	Camembert cheese	[12]
Enterocin CRL35	*E. faecium *CRL 35	Concentrated supernatant	*L. monocytogenes *	Goat milk cheese	[13]
Nisin	*Lc. lactis *CNRZ 150	Starter culture	*L. monocytogenes *	Camembert cheese	[14]
Nisin	*Lc. lactis *TAB 50	Starter culture	*L. monocytogenes *	Semihard cheese	[15]
Lacticin 481	*Lc. lactis* TAB 24	Starter culture	*L. monocytogenes *	Semihard cheese	[15]
Lacticin 3147	*Lc. lactis *DPC 4275	Starter culture	*L. monocytogenes *	Cottage cheese	[16]
Enterocin AS-48	*E. faecalis* TAB 28	Starter culture	*L. monocytogenes *	Semihard cheese	[15]
Enterocin AS-48	*E. faecalis* INIA 4	Starter or adjunct culture	*L. monocytogenes *	Manchego cheese	[17]
Pediocin	*Lc. lactis* MM 217	Starter culture	*L. monocytogenes *	Cheddar cheese	[18]
Pediocin	*Lb. plantarum *WHE 92	Surface sprayed cell suspension	*L. monocytogenes *	Munster cheese	[19]
Pediocin	*Lc. lactis *CL1	Adjunct culture	*L. monocytogenes *	Semihard cheese	[20]
Pediocin	*Lc. lactis* CL1	Adjunct culture	*S. aureus *	Semihard cheese	[20]
Nisin	*Lc. lactis *ESI 515	Adjunct culture	*S. aureus *	Semihard cheese	[20]
